# Dietary Supplementation with *Chrysanthemum morifolium* Ramat cv. ‘Hangju’ Flower Extract Alleviates Skin Photoaging in SKH-1 Hairless Mice

**DOI:** 10.3390/nu18020329

**Published:** 2026-01-20

**Authors:** Yujie Lao, Ruixuan Geng, Mengjie Li, Seong-Gook Kang, Kunlun Huang, Bin Deng, Huiji Zhou, Rong Luo, Tao Tong

**Affiliations:** 1 College of Food Science and Nutritional Engineering, China Agricultural University, Beijing 100083, China; 2 Key Laboratory of Safety Assessment of Genetically Modified Organism (Food Safety), Ministry of Agriculture, Beijing 100083, China; 3 Beijing Laboratory for Food Quality and Safety, Beijing 100083, China; 4 Department of Nutrition and Food Hygiene, School of Public Health, Dalian Medical University, No. 9 West Section Lvshun South Road, Dalian 116044, China; 5 Department of Food Engineering and Solar Salt Research Center, Mokpo National University, Muangun 58554, Republic of Korea; 6 Amway (China) Co., Ltd., Guangzhou 510700, China; 7 Amway (Shanghai) Science and Technology Development Co., Ltd., Shanghai 201203, China

**Keywords:** *Chrysanthemum morifolium* Ramat cv. ‘Hangju’, photoaging, UVB exposure, transcriptome profile, gut microbiota

## Abstract

Background/Objectives: Skin photoaging represents a predominant form of extrinsic aging, characterized by structural and functional impairment of the skin barrier. In severe cases, it may precipitate dermatological diseases and even tumors. Given the prevalence and detrimental effects of skin photoaging, strategies for its effective prevention and mitigation have garnered significant research interest. *Chrysanthemum morifolium* Ramat cv. ‘Hangju’ contains diverse bioactive compounds, including flavonoids, phenylpropanoids, phenolic acids, and polysaccharides, which have been proven to exhibit antioxidant and anti-inflammatory effects. Methods: This study employed a UVB-induced mouse model of skin photoaging to evaluate the potential of dietary supplementation with *Chrysanthemum morifolium* Ramat cv. ‘Hangju’ flower extract (CME) in vivo. Results: In the photoaged skin of female SKH-1 hairless mice, dietary supplementation with CME significantly increased skin moisture content, reduced wrinkle formation, suppressed epidermal hyperplasia, enhanced collagen density, and suppressed the senescence marker expression and DNA damage marker expression. Analysis of the skin transcriptome suggested that CME could alter gene expression patterns and potentially modulate critical signaling pathways involved in skin homeostasis. Moreover, 16S rRNA sequencing indicated that CME mitigated UVB-induced gut microbiota dysbiosis. Conclusions: These preclinical findings reveal the anti-photoaging property of dietary CME supplementation and point to its potential application as a functional dietary supplement for promoting skin health.

## 1. Introduction

The skin serves as the primary defensive barrier that is directly exposed to the external environment, fulfilling critical roles including thermoregulation, waste excretion, sensory perception, and defense against microbial invasion and physical injury. Skin aging is the most visually apparent external manifestation of organismal senescence and constitutes a complex biological process, primarily categorized as intrinsic or extrinsic aging [1]. Intrinsic aging, an inevitable physiological process, is predominantly regulated by genetic factors. Extrinsic aging, on the other hand, is primarily driven by environmental factors, with skin photoaging induced by ultraviolet (UV) radiation representing the leading cause, accounting for approximately 80% of facial aging [2,3]. This process leads to oxidative stress and DNA damage, consequently triggering cellular senescence. Solar UV radiation reaching the surface of the Earth can be classified by wavelength into UVA (320–400 nm, ~95%) and UVB (280–320 nm, ~5%). UVC (200–280 nm) is absorbed and scattered by the ozone layer, preventing it from reaching the ground. Among these, UVB is a major causative factor of direct photodamage to the skin. Although the stratum corneum absorbs most UVB radiation, a portion still penetrates into the dermis, and its high energy can induce significant biological damage [4]. Photoaging compromises skin function and defensive capacity, leading not only to pigmentation, wrinkles, dryness, roughness, and loss of elasticity but also predisposing individuals to various dermatological pathologies, including actinic keratosis, basal cell carcinoma, melanoma, and squamous cell carcinoma [5,6,7]. In 2022, an estimated 330,000 cases of melanoma were newly diagnosed worldwide, and almost 60,000 people died from the disease (source: https://www.iarc.who.int/). Given the substantial public health burden imposed by UVB-induced skin photoaging and related pathologies, it is important to establish effective preventive and interventional strategies and to elucidate their underlying mechanisms.

Current strategies for alleviating or treating skin photoaging primarily involve pharmacological therapies and surgical interventions, which often exhibit limitations in terms of efficacy and potential side effects. For instance, retinoids and similar compounds are frequently poorly tolerated and commonly cause cutaneous irritation or erythema. Surgical approaches, on the other hand, require extended recovery periods and may carry risks of complications [8,9]. Consequently, identifying novel bioactive agents is crucial for combating skin photoaging. With improving living standards and growing health awareness, the concept of “inner beauty” has gained public attention, shifting focus toward dietary supplementation with functional food components to mitigate photoaging [8,10]. Unlike topical applications, this strategy targets the repair of skin cell structure and functionality through endogenous pathways, offering a promising alternative for preventing photoaging. Accumulating evidence indicates that incorporating functional ingredients, including phytochemicals, bioactive peptides, polysaccharides, vitamins, and probiotics into daily nutrition can efficiently ameliorate photoaging-induced skin damage [7,8,10,11,12,13,14].

Gut microbes actively participate in nutrient absorption, preserving the structural integrity of the intestinal mucosal barrier, and modulating the host immune system [15]. Furthermore, gut microbes are closely associated with the aging of extra-intestinal organs, such as the brain, bones, and skin. Research has revealed a bidirectional regulatory mechanism between the gut and skin, referred to as the gut–skin axis, which plays a critical role in preserving skin homeostasis. Dysbiosis of the gut microbiota can influence skin physiology through this axis [16]. For instance, when the intestinal barrier is compromised, gut bacteria and metabolites may translocate into the circulation, accumulate in skin, and disrupt cutaneous homeostasis [17]. It has been reported that *Bifidobacterium* can mediate gut–skin axis homeostasis by restoring microbial balance, alleviating endoplasmic reticulum stress, and promoting the repair of skin barrier damage [18]. Studies have also suggested that diet can change the gut microbiome, thereby exerting either beneficial or detrimental effects on skin health. Consequently, dietary intervention with bioactive compounds to regulate the gut–skin axis represents a promising strategy for mitigating skin photoaging [15].

*Chrysanthemum morifolium*, belonging to the Asteraceae family, is recognized as a medicinal-food homology material by China’s National Health Commission. Its dried capitulum possesses considerable medicinal value, having long been used for the preparation of tea, beverages, and herbal medicines [19,20]. Today, edible chrysanthemum products have become a widely accepted daily dietary practice associated with health consciousness in China. According to the *Pharmacopoeia of the People’s Republic of China (2025)*, *Chrysanthemum morifolium* is classified into five major cultivars. Among these, *Chrysanthemum morifolium* Ramat cv. ‘Hangju’, employed in this study, is the most widely cultivated and introduced variety. Mainly produced in Tongxiang City, Zhejiang Province, *Chrysanthemum morifolium* Ramat cv. ‘Hangju’ is one of the “Eight Famous Herbal Drugs in Zhejiang” (Zhe Bawei), and current studies indicate that it contains diverse bioactive constituents, including flavonoids, phenolic acids, phenylpropanoids, and polysaccharides [21,22,23,24]. A previous study reported that the total flavonoid content in *Chrysanthemum morifolium* ranged from 5.2 to 6.4 mg/g dry weight, whereas the total phenolic acid content ranged from 10.7 to 15.3 mg/g dry weight [22]. Compositional analysis revealed that *Chrysanthemum morifolium* contained total sugar content of 103.1–107.0 mg/g, amino acid content of 1.7–2.4 mg/g, and vitamin C content of 0.336–0.354 mg/g [25]. *Chrysanthemum morifolium* exhibits multifaceted physiological activities, such as antioxidative, anti-inflammatory, cardioprotective, antihypertensive, hypoglycemic, and hypolipidemic effects [19,22,24,26,27,28,29,30,31]. Neatpatiparn et al. demonstrated that *Chrysanthemum morifolium* flower extract exhibits antioxidant, anti-glycation, and anti-collagenase efficacy in vitro [32]. In vivo research revealed that transdermal treatment with bud extracts of *Chrysanthemum morifolium* ameliorated skin moisture loss and collagen degradation in UVB-induced photoaged ICR mice [33]. However, the efficacy of dietary supplementation with *Chrysanthemum morifolium* Ramat cv. ‘Hangju’ flower extract (CME) in alleviating skin photoaging and the underlying mechanisms have not been clarified. In this study, we assessed the impact of dietary CME intake on UVB-induced skin photoaging using skin transcriptomic and gut microbiota analyses to elucidate its mode of action.

## 2. Materials and Methods

### 2.1. Materials

CME (Specification number: KL-CTEP009-001, Batch number: 23120523, flavones content ≥ 5.0%) was purchased from Shanghai Novanat Co., Ltd., Shanghai, China. Silflo silicone was purchased from Beijing Jinhongfan Trading Co., Ltd., Beijing, China. Paraformaldehyde fixative was purchased from Wuhan Servicebio Technology Co., Ltd., Wuhan, China. TRIzol was purchased from American Invitrogen Life Technology Co., Ltd., Carlsbad, CA, USA.

### 2.2. Animal Experiments

Similarly to the established methodologies from earlier studies [10], female SKH-1 hairless mice were used to evaluate the in vivo efficacy of CME against photoaging. Eighteen 7-week-old female SKH-1 hairless mice were procured from Beijing Vital River Laboratories Co., Ltd. (Beijing, China) and housed in specific pathogen-free conditions in an approved animal facility (Approval No.: SYXK (Jing) 2020-0052). Housing conditions included a temperature of 22 ± 2 °C, a 12 h light/dark cycle, and 40–70% humidity. Animal experimental procedures were approved by the Laboratory Animal Welfare and Animal Experimental Ethical Committee of China Agricultural University (AW32504202-4-1, Beijing, China).

After one week of acclimation, the mice were randomly allocated to three experimental groups: Control, UVB, and CME (*n* = 6 per group). Both the UVB and CME groups were subjected to dorsal UVB exposure three times weekly. The UVB irradiation was terminated after 11 weeks. The irradiation system included 3 UVB lamps (TL 40W/12 RS SLV/25, Philips, Amsterdam, The Netherlands) positioned above the dorsal skin surface at a height of 30 cm. UVB intensity was calibrated to 0.225 mW/cm^2^ using a radiometer, with peak sensitivity at 297 nm. An incremental dosing protocol, consistent with established photoaging models was applied: the UVB dose was incremented by one minimal erythemal dose (MED) each week, which was defined as 100 mJ/cm^2^ [34,35]. Based on Equation (1), irradiation sessions during the first week lasted 7.4 min each. The dose was gradually escalated to three MED and kept constant for the rest of the study.

Irradiation time (s) = irradiation dose (mJ/cm^2^)/irradiation intensity (mW/cm^2^) (1)

The standard AIN93G diet was administered to mice in the Control and UVB groups, whereas the CME group was provided with AIN93G containing 0.5% (*w*/*w*) CME (detailed diet composition in Table S1). Throughout the experiment, mice had free access to food and drinking water. Dorsal skin was photographed at regular intervals. At the end of the experimental period, fecal samples were collected in the morning and frozen at −80 °C for further analysis. After a 6-h fast (from 8:00 a.m. to 2:00 p.m.), mice were anesthetized with isoflurane, followed by the replication of skin wrinkles. The mice were subsequently euthanized by cervical dislocation in accordance with ethical guidelines, and dorsal skin tissues were collected for further analysis. One portion of the samples was stored at −80 °C, while the remaining samples were fixed in paraformaldehyde for subsequent processing.

### 2.3. Detection of Skin Moisture Content

After euthanizing the mice, the dorsal skin was taken and cleared of underlying adipose tissue. Skin tissue (about 0.2 g) was first weighed to record wet weight, then oven-dried at 60 °C until the weight stabilized for dry weight determination. The calculation of skin moisture content followed Equation (2):

Skin moisture content (%) = (wet weight − dry weight)/wet weight × 100(2)

### 2.4. Wrinkle Formation Assessment

Prior to sacrifice, the dorsal skin of anesthetized mice was coated with Silflo silicone to generate wrinkle impressions. The replicas were quantitatively evaluated using a PRIMOS CR optical imaging system (Canfield Scientific, Parsippany, NJ, USA), measuring average depth, maximum depth, and total volume of wrinkles.

### 2.5. Hematoxylin and Eosin (H&E) Staining and Masson Staining

After fixation in 4% paraformaldehyde, skin tissues were paraffin-embedded and sectioned at 5 μm. Images were captured using a microscope (NIKON ECLIPSE E100, Tokyo, Japan) after performing H&E and Masson’s trichrome staining. Image J software (version 1.54) was used to quantify epidermal thickness (from H&E images) and collagen density (from Masson’s trichrome images).

### 2.6. Immunohistochemistry Staining of p21 and p53

Paraffin sections (5 μm) were first heated at 65 °C for 2 h and then subjected to antigen retrieval in citrate buffer (pH 6.0) at 121 °C for 2 min. Following serum and H_2_O_2_ blocking, sections were incubated overnight at 4 °C with anti-p21 (1:1000, Wuhan Servicebio Technology Co., Ltd., Wuhan, China) and anti-p53 (1:200, Beijing Bioss Biotechnology Co., Ltd., Beijing, China) antibodies. HRP-conjugated goat anti-rabbit IgG (1:200, Wuhan Servicebio Technology Co., Ltd., Wuhan, China) was applied at 37 °C for 30 min. Hematoxylin and DAB staining was performed, and images were captured under a light microscope. The fraction of positive staining was quantified by Image J (version 1.54).

### 2.7. Skin Transcriptome Sequencing

Skin tissues from the Control, UVB, and CME groups (*n* = 3 per group) were used for total RNA extraction with TRIzol^®^ reagent. RNA concentration and purity were assessed with an ND2000 spectrophotometer (Thermo Fisher Scientific Inc., Waltham, MA, USA) and a 2100 Bioanalyzer (Agilent Technologies Inc., Santa Clara, CA, USA). The Illumina TruSeq™ RNA Sample Preparation Kit (Illumina Inc., San Diego, CA, USA) was employed to construct RNA-seq libraries, which were sequenced with paired-end 150 bp reads on a HiSeq X Ten/NovaSeq 6000 system. After quality filtering and trimming with SeqPrep and Sickle, reads were mapped to the reference genome using HISAT2, and transcript assembly was performed with StringTie.

### 2.8. Differential Expressed Genes (DEGs) and Functional Enrichment Analysis

Transcript abundances and expression levels were quantified using the transcripts per million method in combination with RSEM. DESeq2 was used to perform differential expression analysis, defining DEGs with *p*-adjust < 0.05, fold change ≥1.6 and fold change ≤0.625. Kyoto Encyclopedia of Genes and Genomes (KEGG) pathway enrichment analysis was conducted on the Majorbio Platform (www.majorbio.com), and pathways with *p*-adjust <0.05 were considered significant. Gene Set Enrichment Analysis (GSEA) was also carried out using MSigDB hallmark gene sets (version 6.2) on the Majorbio platform, including gene sets with 15–500 genes. Pathways with significant alterations (*p*-adjust < 0.05) were chosen for further investigation. A Protein–protein interaction (PPI) network was established via the STRING database (https://string-db.org) with a minimum interaction score threshold of 0.4, and hub genes were identified based on connectivity. ChEA3 (https://maayanlab.cloud/chea3, accessed on 18 December 2025) was used to perform transcription factor (TF) enrichment analysis, revealing the top 10 TFs linked to DEGs involved in key signaling pathways.

### 2.9. Fecal DNA Extraction and 16S rRNA Gene Sequencing

Fecal DNA from the Control, UVB, and CME groups (*n* = 3 per group) was extracted using a fecal DNA isolation kit (Beijing Fudean Technology Co., Ltd., Beijing, China) and evaluated with a NanoDrop 2000 spectrophotometer (Thermo Fisher Scientific Inc., Waltham, MA, USA). PCR amplification targeted the V3–V4 regions of the bacterial 16S rRNA gene with primers 338F (5′-ACTCCTACGGGAGGCAGCAG-3′) and 806R (5′-GGACTACHVGGGTWTCTAAT-3′). PCR conditions included an initial denaturation at 95 °C for 3 min, 27 cycles of 95 °C for 30 s, 55 °C for 30 s, and 72 °C for 45 s, and a 10 min final extension at 72 °C, followed by holding at 10 °C (T100 Thermal Cycler PCR thermocycler, BIO-RAD Laboratories Inc., Hercules, CA, USA). Amplicons were subsequently purified, quantified, combined in equimolar amounts, and sequenced in paired-end mode on the Illumina NextSeq 2000 platform (Illumina Inc., San Diego, CA, USA).

### 2.10. Gut Microbiota Data Analysis

After demultiplexing, we processed raw sequences through bioinformatics pipelines and applied the DADA2 plugin in QIIME2 to produce amplicon sequence variants (ASVs). A Naive Bayes classifier, trained with the SILVA 16S database (version 138), was used for taxonomic classification. We calculated α-diversity metrics with Mothur (version 1.30.1) and evaluated β-diversity using Non-metric multidimensional scaling (NMDS) analysis of Weighted UniFrac distance using the Vegan (version 2.5-3) package on the Majorbio platform. We calculated bacterial relative abundances at the phylum and genus levels using Python (version 2.7) to assess microbial community composition. Linear discriminant analysis (LDA) effect size (LEfSe) analysis was conducted to identify taxa differing in abundance across phylum to genus levels, using LDA score > 2 and *p* < 0.05 as thresholds. PICRUSt2 was employed to predict microbial functional profiles, followed by statistical analysis in STAMP (http://kiwi.cs.dal.ca/Software/STAMP, accessed on 18 December 2025). To explore bacterial interactions, we constructed co-occurrence networks based on Spearman correlation coefficients (|coefficient| ≥ 0.6, *p* < 0.05), and visualized network structure and computed topological parameters using Gephi (version 0.10.1).

### 2.11. Statistical Analysis

Data are shown as mean ± SEM. Normality and variance homogeneity were checked before statistical analyses using the Shapiro-Wilk test and Levene’s test, respectively. One-way ANOVA with Dunnett’s multiple comparisons test was used to assess differences among the three groups. GraphPad Prism software (version 10.2.3) was used for all statistical analyses, and significance is indicated as * *p* < 0.05, ** *p* < 0.01, *** *p* < 0.001, and **** *p* < 0.0001.

## 3. Results

### 3.1. Dietary Supplementation of CME Alleviated Skin Dryness and Wrinkle Formation in UVB-Induced Skin Photoaged Mice

**Figure 1:**
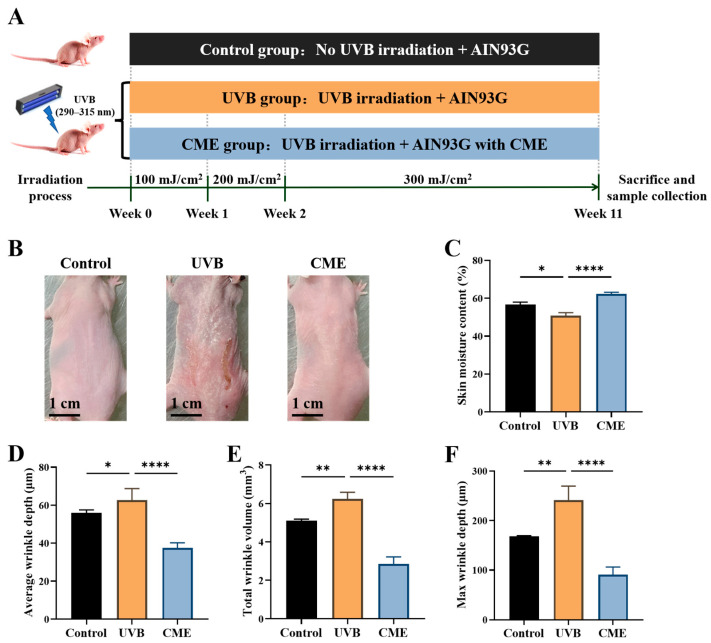
Dietary CME supplementation improves the skin moisture content and alleviates wrinkle formation in SKH-1 hairless mice. (**A**) Overview of experimental design. (**B**) Representative dorsal skin photographs from three groups at the end of the experiment (Scale bar = 1 cm). (**C**) Skin moisture content (*n* = 6). (**D**) Average wrinkle depth (*n* = 3). (**E**) Max wrinkle depth (*n* = 3). (**F**) Total wrinkle volume (*n* = 3). Data are expressed as mean ± SEM. Statistical significance among groups is indicated as * *p* < 0.05, ** *p* < 0.01, **** *p* < 0.0001.

The detailed experimental timeline is presented in Figure 1A. No mortality, behavioral abnormalities, or apparent adverse effects were observed in any of the animals throughout the entire experimental period. There was no significant difference in the average daily food intake between the CME group and the UVB group. Throughout the study, UVB exposure induced prominent cutaneous alterations in mice, including coarse texture, dryness, erythema, and increased wrinkle formation. These adverse phenotypic changes were markedly attenuated by dietary supplementation with CME (Figure 1B). Moreover, UVB exposure resulted in a significant reduction in skin moisture content compared with the control group, whereas CME supplementation effectively restored skin moisture content in photoaged mice (Figure 1C). Prolonged UVB irradiation significantly promoted wrinkle formation, as evidenced by increases in average depth, maximum depth, and total volume of wrinkles. Conversely, CME intervention substantially attenuated these UVB-induced alterations (Figure 1D–F). These results demonstrate that dietary CME alleviates UVB-induced skin morphological changes, moisture content loss, and wrinkle formation.

### 3.2. Dietary CME Decreases Epidermal Thickness and Enhances Collagen Fiber Density in Photoaged Mouse Skin

**Figure 2:**
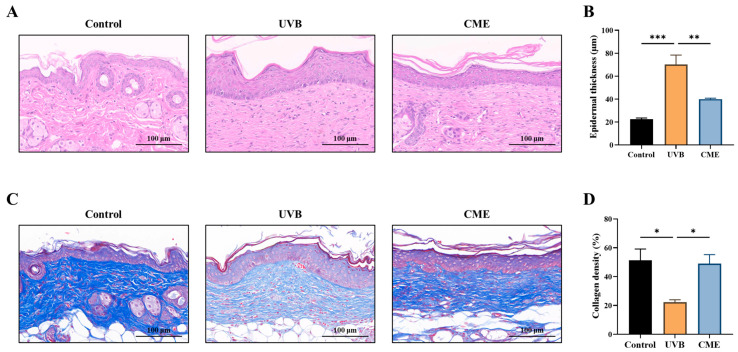
Dietary CME reduces epidermal thickness and enhances collagen fiber density in photoaged mouse skin. (**A**) Representative images of skin histological sections stained with H&E (Scale bar = 100 µm). (**B**) Dorsal skin epidermal thickness. (**C**) Representative images of skin histological sections stained with Masson’s trichrome (Scale bar = 100 µm). Blue color indicates collagen fibers. (**D**) Dorsal skin collagen density. Data are expressed as mean ± SEM. Statistical significance among groups is indicated as: * *p* < 0.05, ** *p* < 0.01, *** *p* < 0.001.

Increased epidermal thickness is a typical histopathological manifestation of skin photoaging [36]. Histopathological examination of skin sections stained with H&E revealed that UVB exposure significantly induced epidermal hyperplasia, whereas dietary supplementation with CME effectively attenuated this pathological thickening (Figure 2A,B). Furthermore, UVB irradiation induces collagen loss in the skin. Masson staining of dermal tissues demonstrated a marked reduction in collagen fiber density following prolonged UVB irradiation (Figure 2C,D). In contrast, dietary CME supplementation significantly restored collagen fiber density in photoaged skin relative to the UVB group (Figure 2C,D).

### 3.3. Dietary CME Inhibits the Expression of Aging Marker p21 and DNA Damage Marker p53 in Photoaged Mouse Skin

**Figure 3:**
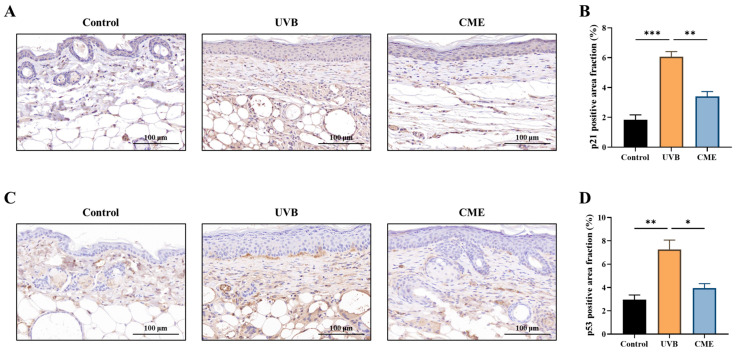
Dietary CME suppresses the levels of aging and DNA damage markers in photoaged mouse skin. (**A**) Representative images of skin histological sections stained by immunohistochemistry for p21 (Scale bar = 100 µm). Brown color indicates p21-positive staining. (**B**) Fraction of p21-positive area in the dorsal skin. (**C**) Representative images of skin histological sections stained by immunohistochemistry for p53 (Scale bar = 100 µm). Brown color indicates p53-positive staining. (**D**) Fraction of p53-positive area in the dorsal skin. Data are expressed as mean ± SEM. Statistical significance among groups is indicated as: * *p* < 0.05, ** *p* < 0.01, *** *p* < 0.001.

To evaluate the anti-aging effects of CME, we examined the expression of p21, a well-established biomarker of UV-induced cellular senescence [35,37]. We found that UVB exposure significantly increased p21 levels in dorsal skin tissues, while dietary CME supplementation markedly attenuated this increase (Figure 3A,B). Given that p53 serves as a direct indicator of UVB-induced DNA damage [38,39], immunohistochemical analysis demonstrated that CME supplementation significantly ameliorated DNA damage in photoaged skin, as evidenced by decreased p53 expression (Figure 3C,D).

### 3.4. Dietary CME Modulates the Skin Gene Expression Patterns

**Figure 4:**
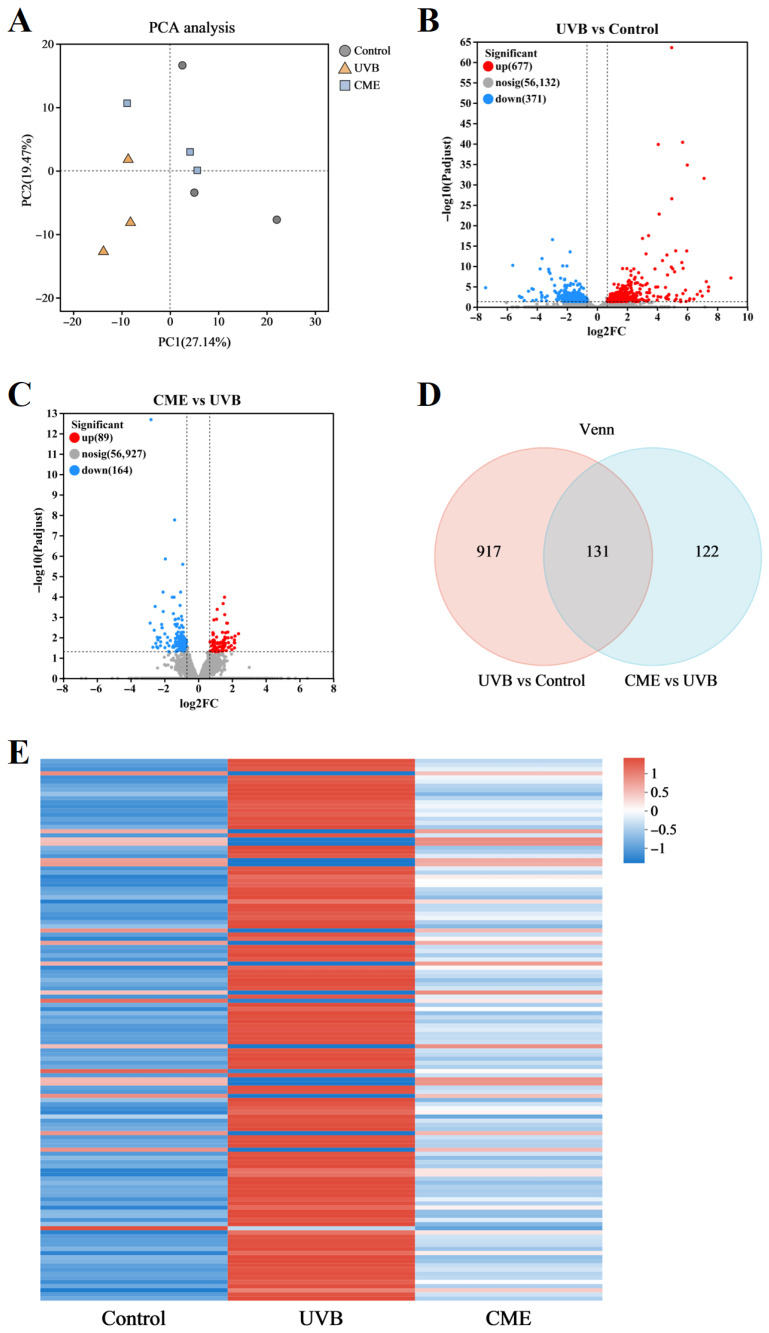
Dietary CME alters skin gene expression patterns. (**A**) PCA of the skin transcriptome. (**B**) Volcano plot of the DEGs in the UVB group mice compared with the Control group mice. (**C**) Volcano plot of the DEGs in the CME group mice compared with the UVB group mice. (**D**) Venn analysis. (**E**) The heatmap of 131 overlapped DEGs. *n* = 3.

To further investigate effects of CME on skin transcriptomic profiles, RNA sequencing analysis was performed on dorsal skin samples from the Control, UVB, and CME groups. Principal component analysis (PCA) indicated clear separation of transcriptomic profiles among the three groups (Figure 4A). Comparison between the UVB and Control groups identified 1048 DEGs, including 677 upregulated and 371 downregulated genes (Figure 4B,C). In the CME group, 253 DEGs were differentially expressed relative to UVB, with 89 upregulated and 164 downregulated (Figure 4B,C). Among these, 131 DEGs were consistently altered in both UVB vs. Control and CME vs. UVB comparisons, as shown by Venn analysis (Figure 4D). A clustering heatmap of the 131 overlapping DEGs suggested that CME treatment partially reversed UVB-induced transcriptomic alterations (Figure 4E).

### 3.5. Dietary CME Downregulates Various Physiological Processes and Signaling Pathways Closely Associated with Photoaging

**Figure 5:**
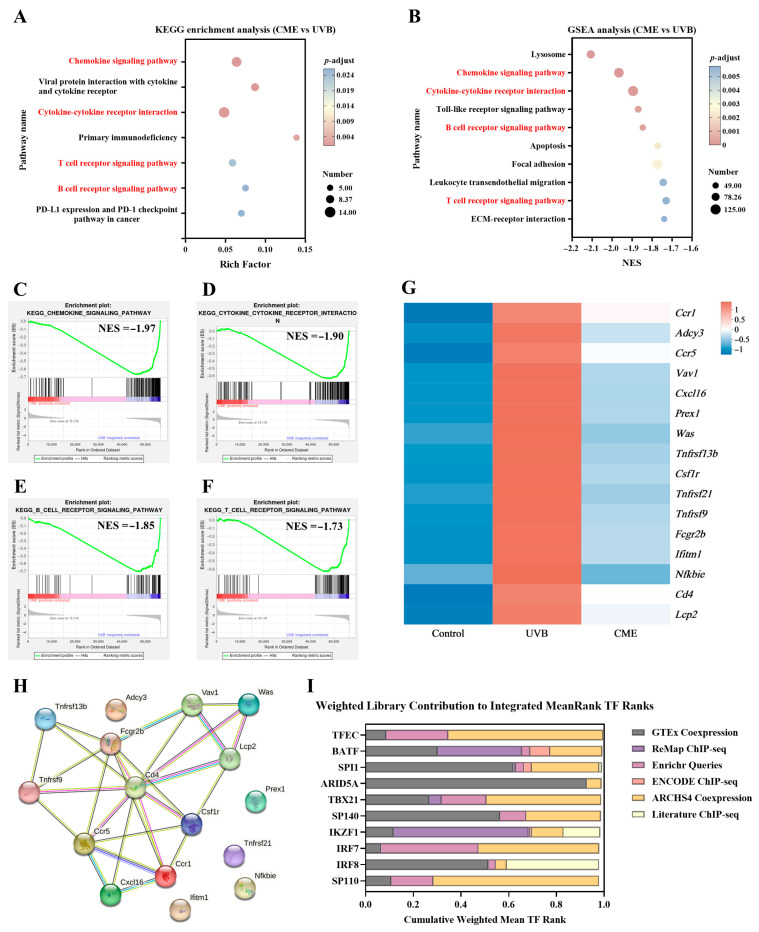
Skin transcriptome enrichment analysis, PPI network analysis, and TF enrichment analysis of the DEGs on key pathways. (**A**) KEGG enrichment analysis of DEGs (CME vs. UVB, *p*-adjust < 0.05). (**B**) GSEA of all genes (CME vs. UVB, *p*-adjust < 0.05). Overlapping pathways are indicated in red font. (**C**) Chemokine signaling pathway enriched by GSEA. (**D**) Cytokine–cytokine receptor interaction enriched by GSEA. (**E**) B cell receptor signaling pathway enriched by GSEA. (**F**) T cell receptor signaling pathway enriched by GSEA. NES, normalized enrichment score. (**G**) Expression heatmap of DEGs associated with the four key signaling pathways among the Control, UVB, and CME groups. (**H**) PPI network of DEGs from the four key pathways. Edge thickness indicates the strength of interaction support. (**I**) Top 10 TFs predicted by ChEA3. *n* = 3.

To explore gene functions and identify key signaling pathways, two complementary analytical methods were employed. KEGG enrichment analysis of the 253 DEGs between UVB and CME groups revealed that dietary CME supplementation significantly modulated multiple signaling pathways in UVB-induced photoaged skin, including chemokine signaling pathway, cytokine–cytokine receptor interaction, primary immunodeficiency, viral protein interaction with cytokine and cytokine receptor, B cell receptor signaling pathway, and T cell receptor signaling pathway (Figure 5A). While KEGG analysis focused on DEGs between UVB and CME groups, GSEA used the full gene expression profile to provide a comprehensive approach to interpret gene expression patterns [40]. GSEA highlighted significantly enriched pathways in the CME vs. UVB comparison that overlapped with KEGG findings, including cytokine–cytokine receptor interaction, chemokine signaling pathway, B cell receptor signaling pathway, and T cell receptor signaling pathway (Figure 5B–F). These four key pathways tended to be downregulated after CME supplementation, indicating that these pathways may be involved in the observed amelioration of skin photoaging. The DEGs involved in these four overlapping pathways, which were significantly regulated by CME, are presented in the heatmap (Figure 5G).

### 3.6. PPI Network Analysis and TF Analysis of the DEGs on Key Signaling Pathways

To determine the main protein-coding genes impacted by dietary CME supplementation, a PPI analysis of all DEGs from the four downregulated signaling pathways was conducted using the STRING 10 database. Hub genes, characterized by high connectivity in the network, are essential for network stability and are considered critical regulatory factors [41]. Using a connectivity threshold greater than 4 as the screening criterion, the PPI network analysis identified five hub genes: *Ccr1*, *Ccr5*, *Csf1r*, *Cd4*, and *Fcgr2b* (Figure 5H). Among these, *Ccr1*, *Ccr5*, *Csf1r*, and *Cd4* were associated with the T cell receptor signaling pathway, cytokine–cytokine receptor interaction, and chemokine signaling pathway, while *Fcgr2b* was linked to the B cell receptor signaling pathway. The exploratory analysis implies that the anti-photoaging effects of dietary CME supplementation may be linked to the regulation of hub genes. To further investigate the potential transcriptional mechanisms underlying the observed variations, TF enrichment analysis was performed using the ChEA3 platform. The top 10 TFs based on the Mean Rank metric were TFEC, BATF, SPI1, ARID5A, TBX21, SP140, IKZF1, IRF7, IRF8, and SP110 (Figure 5I).

### 3.7. Dietary CME Modulates the Gut Microbial Diversity and Structure in Mice with Photoaging

**Figure 6:**
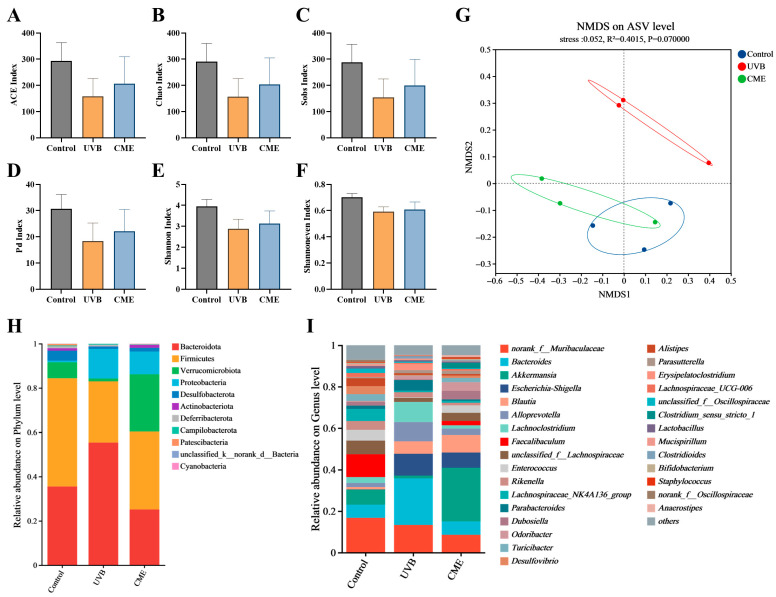
Dietary CME alters gut microbial diversity and structure of gut microbiota in mice with photoaging. (**A**) ACE index. (**B**) Chao index. (**C**) Sobs index. (**D**) Pd index. (**E**) Shannon index. (**F**) Shannoneven index. (**G**) NMDS analysis at the ASV level using Weighted Unifrac distance algorithm. (**H**) Phylum-level gut microbiota composition. (**I**) Genus-level gut microbiota composition. Data for (**A**–**F**) are expressed as mean ± SEM (*n* = 3).

Given the strong connection between the skin and gut microbiota, we further investigated the impact of dietary CME supplementation on the diversity and composition of gut microbiota. Fecal DNA from the three groups was extracted and analyzed by 16S rRNA sequencing. The α-diversity analysis indicated no significant differences between the UVB and CME groups across multiple indexes, including the ACE, Chao, Sobs, Pd, Shannon, and Shannon even indexes (Figure 6A–F). However, NMDS analysis indicated a clear separation in β-diversity between UVB and CME groups (Figure 6G). Notably, the gut microbial structure of the CME group more closely resembled that of the Control group, indicating a beneficial effect of CME on microbial composition (Figure 6G). At the phylum level, Bacteroidota, Firmicutes, and Verrucomicrobiota predominated in the gut communities of both the Control and CME groups (Figure 6H). UVB irradiation reduced the relative abundance of Verrucomicrobiota, increased that of Proteobacteria, and resulted in a higher Bacteroidota/Firmicutes ratio (Figure 6H). Dietary supplementation with CME increased the relative abundance of Verrucomicrobiota, while reducing the relative abundance of Proteobacteria and the Bacteroidota/Firmicutes ratio (Figure 6H). At the genus level, the gut microbiota was primarily composed of *Bacteroides*, *Akkermansia*, and *norank_f__Muribaculaceae* (Figure 6I). The CME group exhibited decreased relative abundances of *Bacteroides*, *Escherichia-Shigella*, *Alloprevotella*, and *Lachnoclostridium* relative to the UVB group, along with increased relative abundances of *Akkermansia*, *Faecalibaculum*, and *unclassified_f__Lachnospiraceae* (Figure 6I). These results indicate that CME intervention alters the gut microbial community in mice with UVB-induced photoaging.

### 3.8. CME Dietary Intervention Changes the Differential Abundant Bacterial and Predicted Function of Gut Microbiota in Photoaged Mice

**Figure 7:**
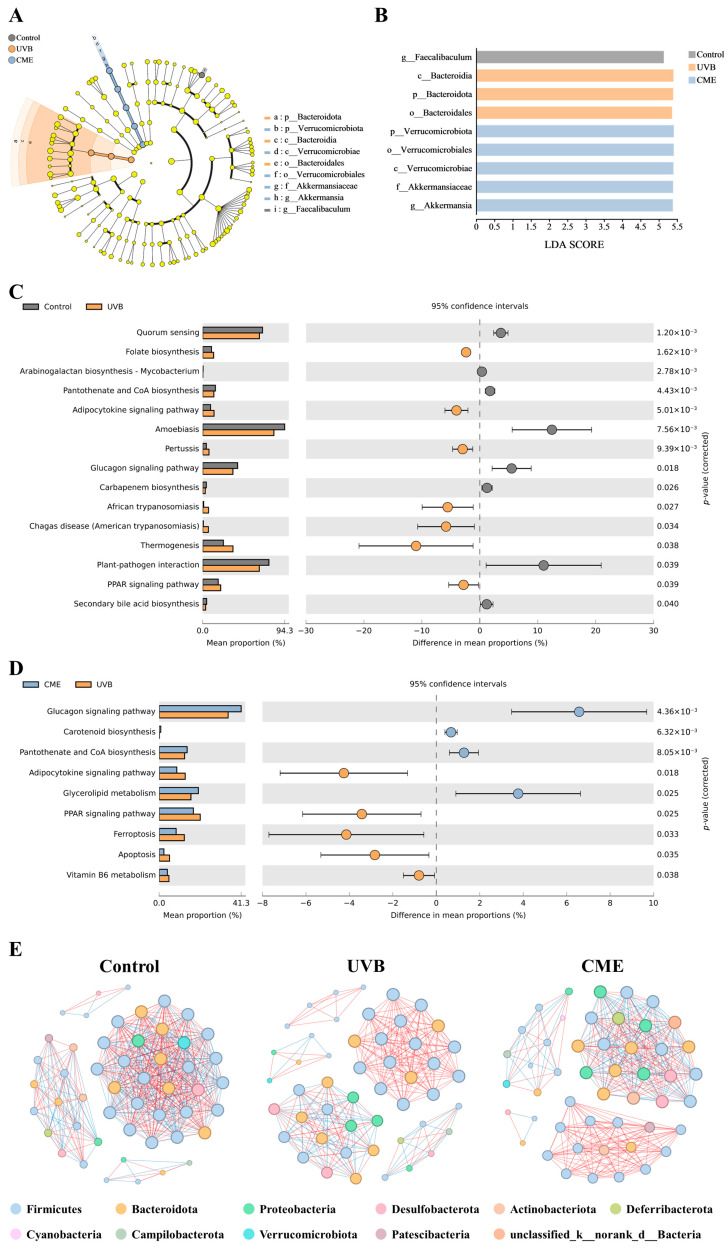
Analyses of differentially abundant bacteria, functional prediction, and co-occurrence network in gut microbiota of photoaged mice after dietary supplementation with CME. (**A**) LEfSe analysis across taxonomic levels (from outer to inner rings: genus, family, order, class, and phylum). (**B**) LDA scores for the bacterial taxa. (**C**) Predicted KEGG functional pathways (Control vs. UVB). (**D**) Predicted KEGG functional pathways (UVB vs. CME). Statistical significance (*p* < 0.05) was assessed by two-tailed Welch’s *t*-test in STAMP. (**E**) Co-occurrence network for three groups. Edges represent significant Spearman correlations between genera (|r| > 0.6, *p* < 0.05). Negative correlations are shown in blue, while positive correlations are shown in red. Node size reflects connectivity, and node color indicates phylum affiliation. *n* = 3.

Differential bacterial taxa in the gut microbiota of the three groups were identified using LEfSe analysis (LDA score > 2). In the Control group, *Faecalibaculum* was identified as a distinctive genus, while Bacteroidia (class level) and Bacteroidales (order level) were distinctive in the UVB group. Notably, the CME group demonstrated significantly enriched taxa including Verrucomicrobiae (class level), Verrucomicrobiales (order level), Akkermansiaceae (family level), Verrucomicrobiota (phylum level), and *Akkermansia* (genus level) (Figure 7A,B).

Predicted functional alterations in gut microbiota after UVB irradiation and dietary CME supplementation were investigated through KEGG database annotation of 16S rRNA gene catalogs. Several pathways were notably affected by UVB exposure and CME supplementation (Figure 7C,D). In the UVB group, the glucagon signaling pathway and pantothenate and CoA biosynthesis tended to be reduced compared to the Control group, while CME supplementation increased these pathways. The adipocytokine signaling pathway and PPAR signaling pathway tended to be enhanced after UVB exposure, and CME supplementation reduced these pathways. These in silico predictions suggested that dietary CME supplementation could reverse the UVB-induced dysfunction of gut microbiota to some degree.

### 3.9. Dietary CME Modifies the Gut Microbiota Co-Occurrence Network in Mice with Photoaging

To elucidate intergeneric relationships within the gut microbiome of CME-supplemented photoaged mice, we constructed co-occurrence networks based on significant correlations (|r| ≥ 0.6, *p* < 0.05). Figure 7E presents the complex microbial co-occurrence networks across the three experimental groups. The results demonstrate that dietary CME supplementation effectively restored the UVB-induced attenuation of microbial network complexity. Quantitative analysis of network topological parameters revealed that the co-occurrence network of the Control group comprised 50 nodes with 472 edges (average degree 18.88, graph density 0.385), whereas UVB exposure reduced these parameters to 49 nodes, 282 edges (average degree 11.51, graph density 0.240) (Table S2). The CME group’s network contained 49 nodes with 347 edges (average degree 14.16, graph density 0.295). Notably, dietary CME supplementation increased the number of edges, average degree, and graph density compared to the UVB group, indicating that CME-treated mice developed microbial networks resembling those of healthy controls. These shifts in co-occurrence patterns suggest that UVB exposure compromises gut microbial ecological connectivity, robustness, and complexity, whereas dietary CME supplementation enhances network stability to promote intestinal health in photoaged mice.

## 4. Discussion

UV radiation, a major environmental factor affecting skin health, exerts complex dual effects on the human body. Regarding its beneficial aspects, moderate UV exposure serves as an essential natural pathway for maintaining vitamin D homeostasis and supporting skeletal health [42]. When the skin is subjected to UVB radiation, the epidermis generates cholecalciferol, a critical precursor to active vitamin D_3_ (1,25-dihydroxycholecalciferol). Furthermore, UV radiation facilitates the release of nitric oxide, which contributes to blood pressure reduction and diminished cardiovascular risk [43]. However, chronic and excessive UV exposure induces various adverse health outcomes, including oxidative stress, collagen degradation, DNA damage, and immune dysregulation, ultimately disrupting normal cellular functions and impairing cutaneous regenerative capacity [10,44]. The negative impacts of sustained UV irradiation on both the skin and internal organs are extensively documented and scientifically validated [45,46]. In this study, the maximum UVB dose was 300 mJ/cm^2^, approximately equivalent to 15 min of sun exposure in Beijing during May, based on Diffey’s solar calculator [47]. In recent years, interventional strategies involving dietary supplementation with botanical extracts to mitigate UV-induced extrinsic skin aging have gained increasing attention [8]. However, investigations into the biological activities of dietary *Chrysanthemum morifolium* Ramat cv. ‘Hangju’ remain relatively limited, and preclinical evidence systematically evaluating the preventive or therapeutic effects of its flower extract against skin photoaging is still scarce. Our findings indicate that dietary supplementation with CME may represent a promising dietary supplement for preventing skin photoaging.

Existing studies have reported that the primary active constituents of *Chrysanthemum morifolium* Ramat cv. ‘Hangju’ are flavonoid and phenolic compounds, such as apigenin, luteolin, chlorogenic acid, and caffeoylquinic acids [22,48,49,50,51]. These components are extensively recognized for antioxidant and anti-photodamage activities. For example, chlorogenic acid has been demonstrated to alleviate UVB-induced photodamage by directly activating SIRT6, which enhances DNA repair capacity [52]. Caffeoylquinic acid derivatives, such as 3,5-dicaffeoyl-epi-quinic acid, protect against UVB-induced photoaging in HaCaT cells by efficiently scavenging reactive oxygen species and suppressing proinflammatory cytokine production [53]. In a *THBS1*^−/−^ mouse model, apigenin significantly inhibited UVB-induced skin tumor formation, as well as inflammatory cell infiltration and cytokine production, in a thrombospondin-1-dependent manner [54]. Both in vitro and in vivo studies have indicated that luteolin alleviates oxidative stress, suppresses matrix metalloproteinase activation, and enhances collagen expression, thereby protecting against UVB-induced skin photoaging [55]. CME also contains polysaccharides and various amino acids, which collectively contribute to its potential nutritional value [27,56]. Our future studies should focus on the detailed compositional separation of CME and the identification and validation of its critical anti-photoaging active components.

*Chrysanthemum morifolium* Ramat cv. ‘Hangju’ possesses both medicinal and edible value and is widely consumed in China for functional beverages and foods [20,22,57]. In a 14-day acute toxicity study, the estimated maximal tolerated dose of *Chrysanthemum morifolium* extract was found to exceed 15 g/kg in both male and female SD rats [58]. A 26-week chronic toxicity study revealed no significant abnormal symptoms or side effects in rats administered doses of 320, 640, and 1280 mg/kg/day of the *Chrysanthemum morifolium* extract [58]. In addition, pharmacokinetic studies investigating oral administration of *Chrysanthemum morifolium* extract to rats at doses of 100, 200, 400, and 12,000 mg/kg reported no significant adverse reactions [59,60]. In our experiments, we observed beneficial effects of dietary supplementation with 0.5% CME on skin photoaging without any apparent adverse reactions, although a comprehensive safety evaluation of CME was not conducted. To assess the human relevance of the selected CME dosage, we employed a dose conversion method based on body surface area normalization [61]. The CME dosage used in this study (0.5% *w*/*w* of diet, i.e., 500 mg/kg/day) corresponds to approximately 2.44 g/day for a 60 kg adult. Future studies are warranted to further evaluate the potential of CME as a standardized dietary supplement for health promotion.

UVB irradiation induces distinctive skin gene expression profile, which can serve as predictive biomarkers for evaluating the anti-photoaging potential of novel candidate compounds. Integrated KEGG enrichment and GSEA, we collectively pinpointed four key signaling pathways that were downregulated following CME supplementation (Figure 5A–F). Among these, T cell receptor signaling pathway, cytokine–cytokine receptor interaction, and the chemokine signaling pathway have been established as critically involved in skin photoaging. Our previous research demonstrated that dietary eugenol supplementation effectively reversed UVB-induced upregulation of cytokine–cytokine receptor interaction in photoaged mouse skin through both KEGG and GSEA analyses [45]. Alafiatayo et al. reported that downregulated DEGs in UV-irradiated human dermal fibroblasts were significantly enriched in cytokine–cytokine receptor interaction and chemokine signaling pathways via KEGG analysis [62]. Similarly, senescent fibroblasts post-UVB exposure exhibited significant enrichment in cytokine–cytokine receptor interaction and T cell receptor signaling pathways [63]. PPI network analysis identified *Ccr1*, *Ccr5*, *Csf1r*, *Cd4*, and *Fcgr2b* as hub genes (Figure 5H). Our exploratory transcriptomic analysis indicated reduced expression levels of these key genes (*Ccr1*, *Ccr5*, *Csf1r*, *Cd4*, and *Fcgr2b*) in the CME group. It is reported that *Ccr1* serves as a potential biomarker for cutaneous inflammatory susceptibility, where its elevated expression may indicate inflammatory status or barrier dysfunction, and has shown positive correlation with acne lesion severity [64]. Both mRNA and protein expression of *Ccr1* are rapidly upregulated following skin injury [65,66]. *Ccr5* has been identified as a biomarker in keloid pathogenesis and atopic dermatitis, potentially mediating inflammatory and fibrotic processes [67]. In metastatic melanoma tissues, mRNA level of *Ccr5* is significantly upregulated, contributing to an immunosuppressive microenvironment [68]. The roles of *Csf1r*, *Cd4*, and *Fcgr2b* in skin biology remain minimally explored. Future investigations should employ inhibitory strategies or genetic knockout models to functionally validate these target genes in photoaging systems.

Emerging evidence indicates that the gut microbiota’s composition and structure are intrinsically linked to skin aging, and intestinal dysbiosis exerts detrimental effects on cutaneous function [8,15]. According to Ghaly et al., UV irradiation affected the fecal microbiome in female C57BL/6 mice [69]. Our earlier studies indicate that prolonged UVB exposure disrupts the gut microbiota in both C57BL/6 and SKH-1 hairless mice [45,70]. In this experiment, we observed that CME ameliorated the phenotypes of skin photoaging and potentially mitigated UVB-induced gut microbial dysbiosis in photoaged mice. Our prior research revealed that 14-week chronic UVB exposure did not notably alter α-diversity but markedly modified β-diversity in female SKH-1 hairless mice [7], which aligns with the current findings (Figure 6A–G). LEfSe analysis identified *Akkermansia*, a genus within the phylum Verrucomicrobiota, as a differentially abundant beneficial microorganism (Figure 7A,B). This bacterium is reportedly capable of producing short-chain fatty acids that modulate carbohydrate metabolism and intestinal immunity, playing pivotal roles in enhancing host immune and metabolic functions [71]. For instance, Seo et al. observed that UVB irradiation significantly reduces the abundance of *Akkermansia* in mouse cecal contents, and an increase in its abundance is positively correlated with improved skin photoaging [72]. Furthermore, oral administration of *Akkermansia muciniphila* can modulate systemic and local immune responses via the gut–skin axis, repair the skin barrier, and significantly alleviate clinical symptoms of atopic dermatitis [73]. In this study, compared with the Control group, the relative abundances of *Akkermansia* were decreased in UVB-irradiated mice, while dietary CME supplementation substantially increased its abundance (Figure 6H,I). Together, our results suggest that the anti-photoaging effects of CME may be associated with modulation of the gut microbiota. However, it should be noted that this study lacks functional validation of microbial changes, such as measuring short-chain fatty acid levels, assessing intestinal permeability, and evaluating systemic endotoxin content, to determine whether these alterations produce corresponding physiological effects. Furthermore, we cannot precisely determine whether alterations in the gut microbiota are the cause or consequence of the attenuated photoaging. Future studies will strengthen mechanistic validation through functional validation experiments and causality testing, such as microbiota depletion or fecal microbiota transplantation studies.

However, the mechanistic insights discussed herein derive from exploratory omics analyses conducted on a limited sample size. Future research requires larger sample sizes to enhance the robustness of omics-related conclusions.

In this study, female SKH-1 hairless mice were utilized to explore the protective effects of dietary CME on skin photoaging and its regulatory impacts on skin transcriptomics and gut microbiota. However, gender is a key biological variable influencing ultraviolet response, systemic metabolism, and gut microbiota. For instance, Parikh et al. found males to be more responsive to solar UV and seasonal changes, with significant metabolic impacts [74]. Reeve et al. observed that UV-exposed hairless male mice developed less intense sunburn inflammatory edema than females [75]. In contrast, Thomas-Ahner et al. indicated that chronic UVB exposure led to more severe skin tumorigenesis, inflammation, and DNA damage in male mice [76]. Kopec et al. reported that female mice exhibit greater resistance to acute UVB-induced skin damage compared to males [77]. Additionally, stress-associated disruptions in gut microbiome diversity and function tend to be more evident in males [78]. Future studies will incorporate both male and female animal models to enable a more comprehensive and accurate evaluation of the effectiveness of dietary interventions in preventing skin photoaging.

## 5. Conclusions

In summary, this study shows that dietary supplementation with CME significantly alleviates skin photoaging in a UVB-induced mouse model. The observed improvements include enhanced skin moisture content, reduced wrinkle formation, suppression of epidermal hyperplasia, increased collagen fiber density, and inhibition of senescence marker expression and DNA damage marker expression. Furthermore, these mitigations under UVB condition were accompanied by changes in gene expression patterns and restoration of gut microbial composition. These findings may provide novel intervention strategies for managing extrinsic skin aging.

## Data Availability

The original contributions presented in this study are included in the article/Supplementary Materials. Further inquiries can be directed to the corresponding author.

## Supplementary Materials

The following supporting information can be downloaded at: https://www.mdpi.com/article/10.3390/nu18020329/s1, Table S1: Composition of the experimental diets (g/kg) fed to mice; Table S2: Global network parameters.
