# Supplementary material for: Dietary Supplementation with Chrysanthemum morifolium Ramat cv. ‘Hangju’ Flower Extract Alleviates Skin Photoaging in SKH-1 Hairless Mice
Source: Nutrients. 2026 Jan 20;18(2):329. doi: 10.3390/nu18020329 (PMC12845440; doi:10.3390/nu18020329)
Supplement: Supplementary file 1 [file nutrients-18-00329-s001.zip › nutrients-4081850-supplementary.pdf]

Table S1. Composition of the experimental diets (g/kg) fed to mice.

Ingredients	Control and UVB groups (AIN93G diet)	CME group (AIN93G diet supplemented with 0.5% CME)
Corn starch	397.486	392.486
Casein	200	200
Maltodextrin	132	132
Sucrose	100	100
Soybean oil	70	70
Cellulose	50	50
Mineral mix[1]	35	35
Vitamin mix[2]	10	10
L-Cystine	3	3
Choline bitartrate	2.5	2.5
tert-Butylhydroquinone	0.014	0.014
CME	-	5

[1] Mineral mix: AIN93G–Mineral mix.

[2] Vitamin mix: AIN93G–Vitamin mix.

Table S2. Global network parameters.

Parameters	Control group	UVB group	CME group
Number of nodes	50	49	49
Number of edges	472	282	347
Average degree	18.88	11.51	14.16
Graph density	0.385	0.240	0.295
